# Role of germline variants in the metastasis of breast carcinomas

**DOI:** 10.18632/oncotarget.28250

**Published:** 2022-06-30

**Authors:** Ángela Santonja, Aurelio A. Moya-García, Nuria Ribelles, Begoña Jiménez-Rodríguez, Bella Pajares, Cristina E. Fernández-De Sousa, Elísabeth Pérez-Ruiz, María del Monte-Millán, Manuel Ruiz-Borrego, Juan de la Haba, Pedro Sánchez-Rovira, Atocha Romero, Anna González-Neira, Ana Lluch, Emilio Alba

**Affiliations:** ^1^Instituto de Investigación Biomédica de Málaga (IBIMA), Hospitales Universitarios Regional y Virgen de la Victoria de Málaga, Spain; ^2^Laboratorio de Biología Molecular del Cáncer, Centro de Investigaciones Médico-Sanitarias (CIMES), Universidad de Málaga, Málaga, Spain; ^3^Departmento de Biología Molecular y Bioquímica, Universidad de Málaga, Málaga, Spain; ^4^Unidad de Gestión Clínica Intercentro de Oncología, Instituto de Investigación Biomédica de Málaga (IBIMA), Hospitales Universitarios Regional y Virgen de la Victoria de Málaga, Málaga, Spain; ^5^Centro de Investigación Biomédica en Red de Oncología, CIBERONC-ISCIII, Madrid, Spain; ^6^Medical Oncology Service, Hospital Costa del Sol, Marbella, Málaga, Spain; ^7^Instituto de Investigación Sanitaria Gregorio Marañón, Universidad Complutense, Madrid, Spain; ^8^Medical Oncology Service, Hospital Virgen del Rocío, Sevilla, Spain; ^9^Biomedical Research Institute, Complejo Hospitalario Reina Sofía, Córdoba, Spain; ^10^Department of Oncology, Complejo Hospitalario de Jaén, Jaén, Spain; ^11^Molecular Oncology Laboratory, Hospital Clínico San Carlos, IdISSC, Madrid, Spain; ^12^Human Genotyping-CEGEN Unit, Human Cancer Genetics Program, Spanish National Cancer Research Centre (CNIO), Madrid, Spain; ^13^Department of Oncology and Hematology, Hospital Clínico Universitario, Valencia, Spain; ^14^INCLIVA Biomedical Research Institute, Universidad de Valencia, Valencia, Spain; ^*^These authors contributed equally to this work

**Keywords:** breast cancer, germline variants, epistasis, network analysis, seed and soil

## Abstract

Most cancer-related deaths in breast cancer patients are associated with metastasis, a multistep, intricate process that requires the cooperation of tumour cells, tumour microenvironment and metastasis target tissues. It is accepted that metastasis does not depend on the tumour characteristics but the host’s genetic makeup. However, there has been limited success in determining the germline genetic variants that influence metastasis development, mainly because of the limitations of traditional genome-wide association studies to detect the relevant genetic polymorphisms underlying complex phenotypes. In this work, we leveraged the extreme discordant phenotypes approach and the epistasis networks to analyse the genotypes of 97 breast cancer patients. We found that the host’s genetic makeup facilitates metastases by the dysregulation of gene expression that can promote the dispersion of metastatic seeds and help establish the metastatic niche—providing a congenial soil for the metastatic seeds.

## INTRODUCTION

The metastatic dissemination of the disease causes the overwhelming majority of cancer-related deaths, yet this enormously complex process remains poorly understood. The different models and unifying concepts that explain metastasis cannot yet explain the adaptive programs that allow tumour cells to thrive in distant tissues and, therefore, the biological and clinical observations associated with metastasis [1, 2].

The metastatic cascade requires the cooperation of tumour cells with different cells of the rest of the organism. Carter et al., showed how the genetic background (i.e., the inherited polymorphisms carried in the germline) could influence the somatic evolution of a tumour in at least two ways: by determining the site of tumourigenesis and modifying the likelihood of acquiring mutations in specific cancer genes [3]. Furthermore, genes are not expressed in isolation but promote the expression of other genes in a coordinated pattern. Therefore, the tendency of a tumour to metastasise may be determined by coordinated changes in gene expression. Studies based on many prognosis signatures [4–7] show that the coordinated gene expression in most cells present early in tumourigenesis—i.e., gene regulation—often determines tumour biology.

Animal models and epidemiological studies suggest that the risk of developing metastasis after breast cancer diagnosis depends on the characteristics of the tumour and germline gene variants [8, 9]. Furthermore, Xu et al., have shown how germline variants of natural killer cells in the tumour immune microenvironment can sway metastasis risk in several cancers [10]; they have also identified germline genomic patterns that contribute to cancer progression and metastasis [11].

Germline variants predate and complement tumour cells’ somatic variants (mutations). The tumour acquisition of further mutations empowers its cells to disseminate and proliferate in a distant tissue (i.e., metastatic seeds), and the host’s genetic makeup promotes metastasis by providing a congenial soil [12]. Germline genetic variants that facilitate metastasis tend to be spread across the genome and interact with one another. In complex traits such as metastasis, multiple genetic influences are responsible for moderate differences in survival. Variants with a single, potent effect on the phenotype are rare. Therefore, the metastatic phenotype depends on accumulating weak effects on a substantial fraction of the genes that comprise the regulatory pathways driving metastasis (Boyle et al., 2017).

Detecting these collectives of interacting variants, each with modest effect size, requires substantially greater research effort than the individual strong-effect variants usually studied [13]. The extreme discordant phenotypes approach based on comparing high-risk healthy individuals—and therefore likely to bear the genetic variants that protect them from disease—with sick, low-risk individuals likely to bear the genetic variants that predispose them to that disease. This approach assumes that the patients at both ends of the disease spectrum are the most informative and, therefore, requires fewer patients to genotype and increases the statistical power of gene association studies [14].

Epistasis networks constitute a novel technique to identify genetic variants associated with a disease that accounts for the heritability of complex traits—traits for which the interactions among many genes control the variations between individuals [13, 15]. Metastasis is such a complex process that metastasis susceptibility is probably due to complex allelic combinations of germline variants [16]. Therefore, metastasis is the perfect ground to deploy epistasis networks since they rest on the idea that the synergistic interactions among many genetic variants, each with a moderate individual effect, determine the disease susceptibility. Epistasis emphasises that the synergistic interactions among genetic variants determine their effect on the phenotype; that is, the effect of one genetic variant on a given trait depends on the genotype of many other variants affecting the trait [17]. Therefore, epistasis has the potential to characterise the network of interactions among genetic variants that shapes the genetic architecture of metastasis [18].

Agarwal et al., proposed that germline variants complement somatic mutations as breast cancer drivers—although germline polymorphisms affect critical biological processes required for breast carcinogenesis, their action is not enough for cancer initiation. Pre-existing germline variants could determine the subsequent somatic mutations required for cancer initiation [19]. Our work is based on a similar hypothesis: what constitutes a somatic event that drives metastasis in breast cancer is conditional on the collection of germline variations in the patient. Therefore, in this work, we have analysed the host factors (i.e., germline variants) that contribute to the susceptibility to metastasis in breast cancer patients. According to the extreme discordant phenotypes approach, our analysis framework is based on genome-wide genotyping of a small set of patients at the extreme of the metastasis susceptibility distributions—low-risk individuals who unexpectedly relapse within five years of follow-up and high-risk patients without relapse. We performed an epistasis network analysis to detect the variants that take part in metastasis by modulating the effect of other variants. We selected the genes that harbour these germline variants based on their role in the regulation of metastasis. We found several gene candidates through which the host’s genetic makeup contributes to metastasis.

## RESULTS AND DISCUSSION

### Characteristics of the study and patients

We performed genome-wide genotyping in a cohort of 97 breast cancer patients that showed metastatic extreme discordant phenotypes: 34 good prognosis cases (with tumours smaller than 2 cm and no lymph nodes affected who relapsed within five years after surgery) and 63 poor prognosis cases (patients with more than ten lymph nodes affected who did not relapse within five years after surgery). This design has the advantage of reducing the phenotypic heterogeneity of the cohort, resulting in a potential enrichment of germline variants associated with metastasis predisposition. The median age at diagnosis was 50 years (range 29–89), and about half of the patients were postmenopausal at diagnosis (55%). Patients had mainly tumours with histological grade 2 (50%), hormone receptor-positive (72%) and HER2 negative (70%). Most of them received adjuvant therapy: chemotherapy (71%), hormonotherapy (68%), or radiotherapy (78%). None of the patients stopped the treatment unless they had progression. Supplementary Table 1 shows the characteristics of patients in the good and poor prognosis groups. We found a similar proportion of immunohistochemical subtypes in both cohorts (~55% luminal, ~20% HER2+ and ~15% triple-negative). Therefore, the prognosis risk in each group was likely due to clinical size and lymph node involvement and not enrichment in different biological subtypes. Patients from the poor prognosis cohort received more adjuvant treatment than patients from the good prognosis group, as expected by their clinicopathological characteristics (tumour size and lymph node involvement).

### Epistatic interactions unveil genes contributing to metastasis susceptibility

We obtained 2016 SNPs from our genome-wide genotyping of extreme discordant phenotypes ranked by SNPRank (see SNPs with SNPRank score > 0.5 in Table 1 and the 2016 SNPs in Supplementary Table 2). According to their minor allele frequencies (MAF) obtained from the 1000 Genomes Project, these SNPs are frequent in the population (91% of the SNPs had MAF greater than 5% and thus can be considered very common) and not different from what is expected by chance (Chi-square test, *p*-value = 0.76). However, SNPs at the top of the rank (i.e., those in Table 1) have lower MAF than the complete list of SNPs (median 0.09; IQR: 0.05, 0.17; Welch’s *t*-test *p*-value < 0.0001). Some of these top SNPs are in or near genes associated with either enhanced metastatic dissemination or with reduced metastatic ability–see, for instance, *PIK3C2B* [20], *ZAP70* [21] and *VIP* [22].

**Table 1:** Top SNPs ranked by SNPrank score

SNP ID	Gene	Chromosome	SNP Rank score	SNP location	MAF
rs11139965	RN7SKP242	9	0.5898	intergenic	0.170
rs67242866	LINC01362	1	0.5784	intergenic	0.028
rs199830092	PIK3C2B	1	0.5749	intron variant	NA
rs72951131	ZAP70	2	0.5587	intron variant	0.183
rs34000182	MIR5689HG	6	0.5485	intron variant	0.053
rs61776380	RNU6-830P	1	0.5444	intergenic	0.065
rs778902	ISCA1P2	1	0.5378	intergenic	0.098
rs2501357	C1orf204	1	0.5360	intron variant	0.306
rs742635	ABTB2	11	0.5349	intron variant	0.085
rs16927008	CLVS1	8	0.5324	intron variant	0.027
rs4849127	IL1B	2	0.5257	downstream gene variant	0.125
rs41263676	C1orf21	1	0.5251	3’UTR variant	0.131
rs11692741	MYT1L	2	0.5211	intron variant	0.180
rs77142354	MIR4300HG	11	0.5165	intron variant	0.039
rs77162747	ZNF767P	7	0.5144	intergenic	0.072
rs77169575	MAML3	4	0.5142	intron variant	0.198
rs9994379	ELOVL6	4	0.5136	intron variant	0.034
rs13421497	PRKCE	2	0.5134	intergenic	0.073
rs1197934	LINGO2	9	0.5128	intron variant	0.112
rs75394800	EML6	2	0.5115	intron variant	0.033
rs3799142	VIP	6	0.5039	downstream gene variant	0.172

Abbreviation: MAF: minor allele frequency.

Previous studies that tried identifying germline variants associated with breast cancer survival did not find any that reached genome-wide significance [23–25]. In line with these results, we did not find any germline variants that showed a strong single-effect on the predisposition to metastasis at the required GWA statistical significance (*p*-value < 5 × 10^*−*8 ^). Similarly, when looking for these 2016 SNPs in dbSNP, only ten were previously associated with breast cancer two were associated with breast cancer risk, and none were associated with metastasis. This underannotation could be because dbSNP includes SNPs found in tumour/normal tissue, i.e., somatic variants, while we are investigating germline variants that have not been described previously. Another possibility is that none of these variants has a strong single-effect, as shown by previous GWAS. Thus, our genotype analysis suggests that germline variants do not affect the susceptibility to metastasis by acting individually on a few genes; they act in coordination over many genes.

We looked for germline variants that affect the susceptibility to metastasis through epistatic interactions to study this pervasive action of germline variants over many genes. These are statistical interactions between loci in their effect on a trait such that the impact of a particular single-locus genotype depends on the genotype at other loci. Therefore, the germline variants in our cohort might affect genes that are associated with regulatory networks and pathways, driving the susceptibility to develop metastasis. We modelled the gene epistasis network that encodes the susceptibility to develop metastasis in our cohort and identified the core genes that direct the susceptibility to metastasis by the community centrality measure.

**Figure 1:**
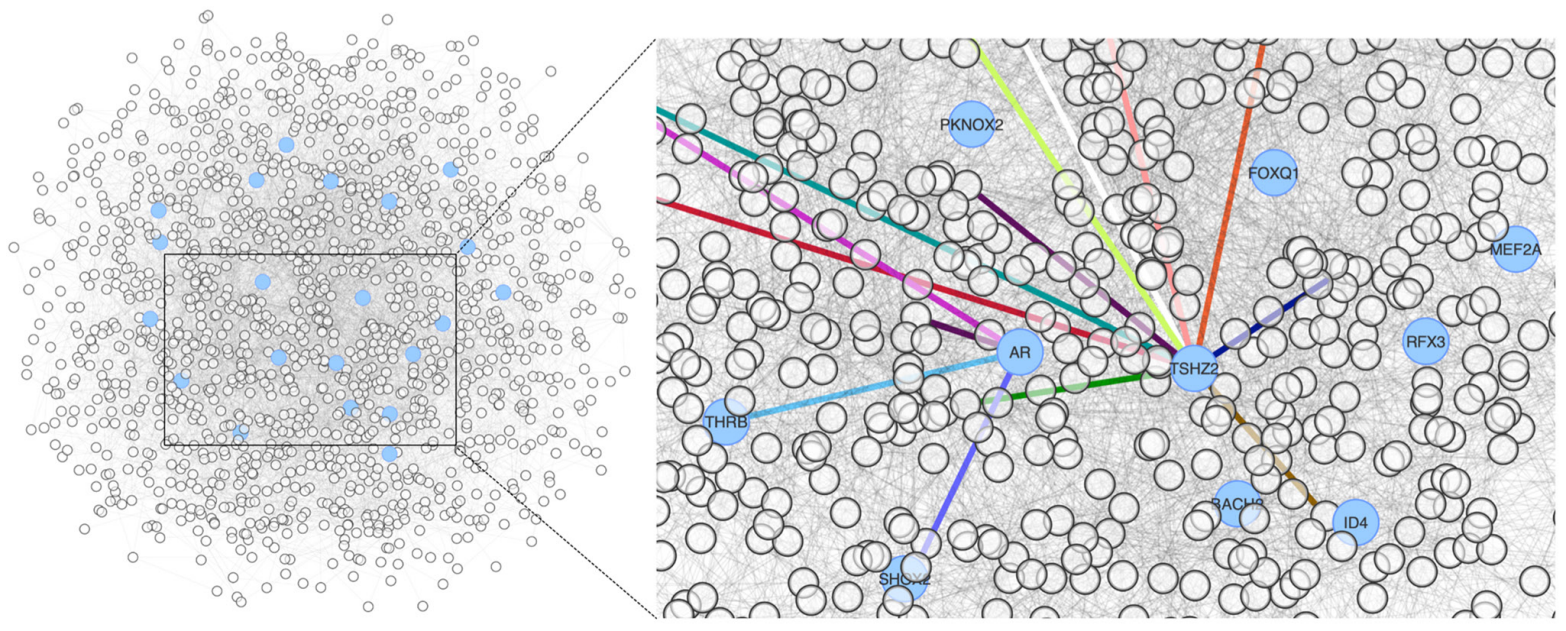
Epistasis network encoding the susceptibility to metastasis in our cohort. The genes with high community centrality are represented in blue. The right panel highlights the participation of two community-central genes in several communities by the colour of their links.

The epistasis network contains 1428 genes and ca. 5600 links among them. It is a large and dense network (i.e., there are many links among genes), which further emphasises the polygenic nature of the germline contribution to a complex trait such as metastasis. It is also a small-world network, meaning that most genes can be reached from every other gene in a few steps and that genes are tightly interconnected, forming communities. The sheer number of nodes and edges obscure essential features such as the nodes that support the integrity of connections—around which the network is organised. The centrality of these nodes is an indicator of a particular node’s relevance to the network’s large-scale structure, which helps us prioritise nodes and identify the essential genes in the epistasis network. The epistasis network is modular, i.e., it is formed of communities that group together highly interconnected nodes representing related genes that work together. The interactions within a community are somehow autonomous from interactions in other communities; thus, in the epistasis network, each community embodies a different aspect of the susceptibility to metastasis. In such a network, the nodes that participate in several communities partake in most interactions throughout the network, connecting different communities otherwise isolated and maintaining the global network structure. These are the core genes expected to play a direct role in metastasis by their influence on many other genes—which germline variants might also perturb—that either promote the migration of tumour cells or favour the seeding of cells disseminated from the primary tumour in target tissues. Figure 1 shows the epistasis network and illustrates the community centrality using AR and TSHZ2, two examples of genes influencing the susceptibility to metastasis because they connect several communities in the epistasis network.

We found that the top 10% of community-central genes are overrepresented in KEGG pathways (over-representation test; multiple comparisons adjusted and false discovery rate controlled), such as the interaction with extracellular matrix receptors (KEGG: hsa04512; *q*-value = 0.018) and the establishment of cell-extracellular matrix contact points (KEGG: hsa04510; *q*-value = 0.017). This implies that the genetic variants of breast cancer patients who tend to develop metastasis affect genes mechanistically involved in metastasis.

The preliminary analysis of our genotyping data suggests that the community-central genes integrate the communities effectively in the topology of the epistasis network. Therefore, the community-central genes contribute extensively to metastasis by influencing many other genes implicated in metastasis.

### Genes influence breast cancer metastasis through gene regulation

According to the seed and soil hypothesis, we postulate that the community-central genes partake in metastasis either by being part of the metastatic seeds—expressed in the tumour or its microenvironment—or by priming the congenial soil—i.e., they are expressed in non-tumour tissue. Our analysis of the epistatic interactions suggests that germline variants affect genes expressed either in the tumour and its microenvironment or target tissues, making the primary tumour more prone to develop metastasis. Since a tumour’s capability to metastasise depends on the coordinated gene expression present early in tumour development, we also postulate that the genes harbouring germline variants will be critical players in gene regulation. To analyse the regulatory role of the community-central genes, we have modelled a gene regulatory network for metastasis in breast cancer.

We modelled a metastasis gene regulatory network by expanding a set of metastasis genes on a breast cancer gene regulatory network that contained 254 TFs, 3178 target genes and 9414 TF-target interactions. These metastasis genes are dysregulated in metastatic tumours and responsible for the dedifferentiation to cancer stem cells, for stem cells are crucial in establishing the premetastatic niche—they mobilise and eventually arrive in and manipulate the secondary microenvironment in sites that will become metastases [26, 27]. The network comprises 142 transcription factors that regulate the expression of 373 target genes. Although the network is dense, connections are not evenly distributed, for the metastasis regulatory network is scale-free. That means that there are a few nodes with many connections, and most nodes have very few connections. For example, the median number of connections for a gene in the metastasis regulatory network is 4, but 9 genes (all of them are transcription factors) have more than ten times the median number of connections and 81% of the genes have lower than twice the median number of connections. Since all the connections come from TF and end up in target genes, the TF regulating many target genes have the most outgoing connections. In contrast, genes subjected to extensive regulation have many incoming connections in our metastasis regulation network. This handful of TF and target genes holds a privileged position in gene regulation.

The metastasis regulatory network is modular. That means that genes interact more closely within the community than with other parts of the network. Communities in a network tend to be associated with a biological function. Therefore, in our metastasis regulatory network, the communities are associated with processes and biological functions related to metastasis.

**Figure 2:**
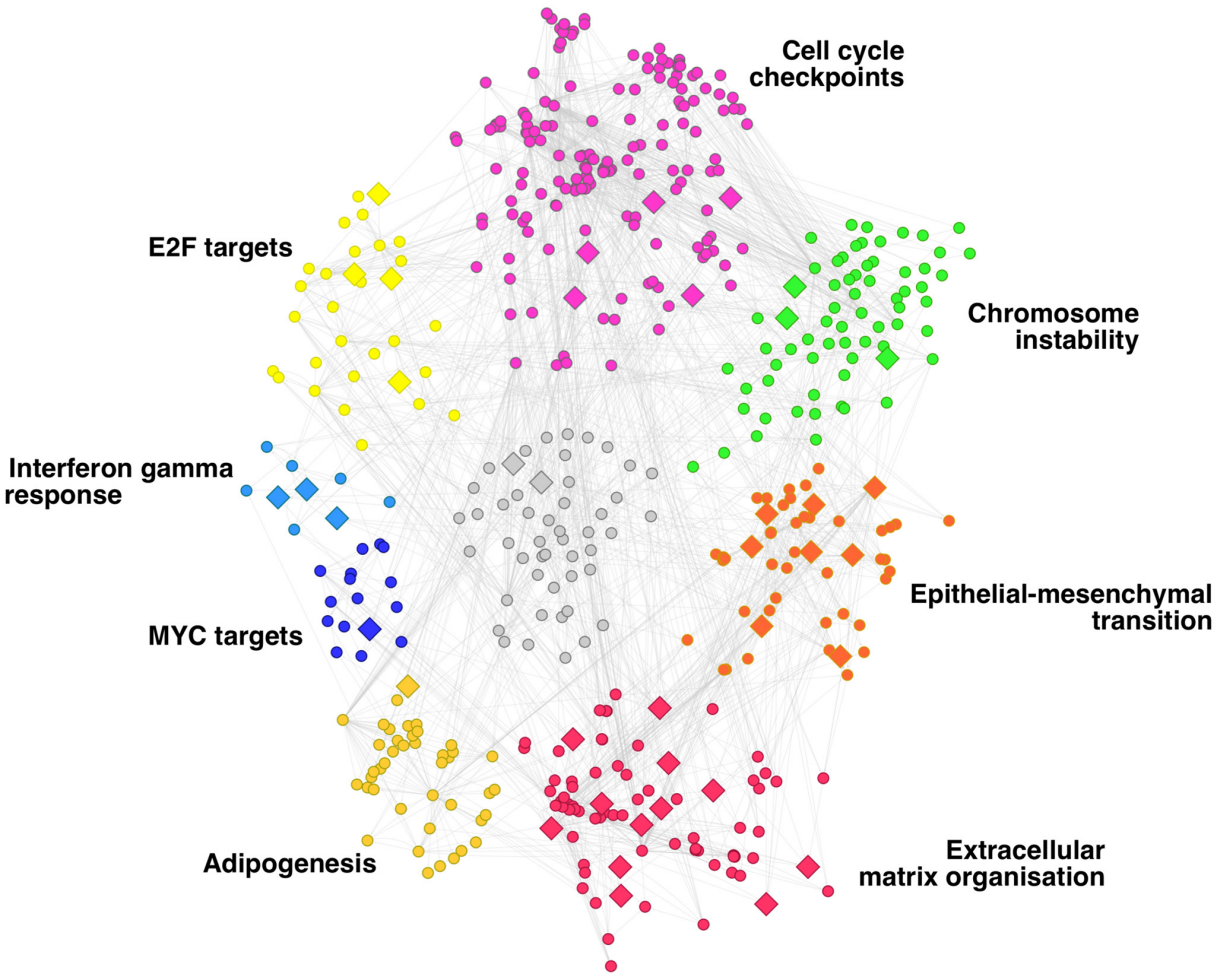
Gene regulatory network of breast cancer metastasis. Network communities are depicted in different colours and annotated according to the enriched functions of their genes.

Figure 2 illustrates the composition of the network in communities. We can see that the yellow community (31 genes) is enriched in genes regulated by E2F—which promotes metastasis in breast cancer [28].

The genes regulated by MYC form the blue community (16 genes), whereas the dark yellow (43 genes) and purple (67 genes) communities are formed by genes associated with adipogenesis and cell cycle checkpoints, respectively. Both processes are relevant in metastasis. Adipogenesis is related to metastasis in triple-negative breast cancer through different mechanisms (Oshi et al., 2021). The dysregulation of genes involved in the cell cycle checkpoints is associated with aggressive cellular behaviour, including invasion- and metastasis-associated changes [29]. The genes involved in the response to Interferon gamma (IFN-γ) signalling arrange in the small light blue community (9 genes). The intensity of the IFN-γ signalling can describe the pro-metastatic role of tumours since tumours treated with low-dose IFN-γ acquired metastatic properties while those infused with high dose led to tumour regression [30].

Chromosome instability is another tumour feature that leads to the metastatic phenotype [31–33]. Our metastasis regulatory network captures chromosome instability in the green community (72 genes). Finally, the two processes are more clearly associated with metastasis, the epithelial-mesenchymal transition (EMT) and the extracellular matrix’s remodelling. These two processes are fundamental for the metastasis hallmark of motility and invasion (Welch and Hurst, 2019). They are present in our regulatory network across the red (55 genes) and orange (48 genes) communities, respectively.

More (smaller) communities on the network are not highlighted in Figure 2 because they are loosely related to metastasis. These results show that we have modelled a gene regulatory network that encodes the complex regulation of breast cancer metastasis.

Since inherited genetic variants tend to associate with complex phenotypes through the regulation of gene expression [34], we assessed whether the genes with germline variants might regulate the expression of tumour genes, thus influencing metastasis. We have used the gene regulatory network focused on metastasis in breast cancer to determine genes harbouring germline variants of metastasis susceptibility that effectively participate in metastasis. We mapped our community-central genes onto the metastasis regulatory network and analysed their relevance in the network topology, which indicates their importance in metastasis. Thirty-nine genes out of the 1428 genes present on the epistasis network map to the metastasis regulatory network. Sixteen of them are TFs that target 23 genes in the metastasis regulatory network. These transcription factors tend to have larger regulons in the breast cancer gene regulatory network than the rest of the transcription factors in the network (average regulon size of 40 and 30, respectively). The 23 target genes are not more extensively regulated than the rest of the target genes in the network; on average, three transcription factors regulate each gene.

The 39 genes that have germline variants associated with metastasis and that participate in the regulation of metastasis tend to be in the communities highlighted in Figure 2. Half of the genes are in the communities associated with the EMT and the extracellular matrix reorganisation; six genes are in the community associated with the alterations in cell cycle checkpoints. These processes are essential for the dispersion of the metastatic seeds and for establishing the pre-metastatic niche.

The TFs and the targets of regulation that bear germline variants could participate in breast cancer metastasis by regulating many other genes. Therefore, they are representative of the host genetic makeup that makes some breast cancer patients more susceptible to develop metastasis. We postulate that these regulators and regulated genes influence the predisposition to develop metastasis in breast cancer patients, and thus we termed them metastasis influence genes. The rest of the paper focuses on how these 39 genes partake in metastasis.

The metastasis regulatory network has a bow-tie structure, i.e., a structure in which the genes that form a tightly interconnected inner core facilitate the effective communication between the genes in the periphery of the network, the TFs and the genes under their regulation. The metastasis influence transcription factors are significantly overrepresented on the bow-tie core of the network (one proportion z-test, *p*-value < 0.05). Of the five transcription factors that harbour germline variants of metastasis susceptibility, TSHZ2 is an important regulator that participates in breast cancer and metastasis [35]. Our metastasis influence genes are significantly central (one proportion z-test, *p*-value < 10^*−*6 ^), which means that they tend to be relevant in the network, i.e., the network revolves around these genes. The relevance in the network translates to importance in the phenotype encoded in the network [36]. Therefore, our network analysis suggests that we have selected the genes that contribute most effectively to metastasis among those that have germline variants associated with metastasis susceptibility.

### Metastasis influence genes are expressed across all breast cancer subtypes and in metastatic breast cancer cell lines

Breast cancer molecular subtypes are associated with survival and patterns of distant metastasis. For example, the luminal A subtype is associated with the longest survival times, followed by luminal B, HER2-enriched and Basal-like [7, 37]. Therefore, the expression in breast tumours of our metastasis influence genes could be directed by the breast cancer subtype.

We have tested the expression of the 39 metastasis influence genes in the breast cancer cohort of the TCGA. This cohort has gene expression data for 1100 tumour samples and 112 control samples; we used both tumour and control samples to assess whether each community-central gene is expressed in breast cancer. We calculated the proportion of tumour samples for each gene in which the gene is differentially expressed compared with control samples. The gene is expressed in a subtype if the tumour expression index is higher than 0.35 in that PAM50 subtype (see Methods).

The metastasis influence genes are expressed in all the molecular subtypes, except for the transcription factors EN1 and AR and the regulated gene SMARCD3. EN1 and AR are expressed only in basal-like tumours, and SMARCD3 is not expressed in luminal-A and normal-like tumours (Supplementary Tables 3 and 4 have the Kaplan-Meier *p*-values for the metastasis influence genes and their regulons, respectively). Even though the molecular subtypes can have different tendencies to produce metastasis and have an assorted pattern of association with distant metastasis-free survival, our results apply to breast cancer regardless of its subtype since all the genes of interest are expressed in all the subtypes—with the caveat of EN1, AR and SMARCD3 mentioned above.

This result agrees with the idea that tumour size and lymph node involvement have a more substantial effect on metastasis than the molecular subtype of the tumour. These two factors are the most relevant in the prognosis of localized breast cancer even when the hormone receptors and HER2 status were not assessed. Carter et al., [38] showed that 5278 patients with tumours smaller than 2 cm and no lymph node involvement had a 5-year survival of 96.3%, suggesting that the metastasis-free survival was even higher. Furthermore, the prognosis of patients with more than ten metastatic axillary lymph nodes had a five-year disease-free survival of 30–39%, independently of the adjuvant treatment received and their ER status [39]. Therefore, the predictive value of the subtype (and its importance in developing metastasis) is less relevant than the tumour size and the number of lymph nodes involved, which are the criteria we have used to design our cohort of patients. That is why the metastasis influence genes we have found are evenly expressed in all the subtypes.

We compared the expression of the metastasis influence genes in metastatic vs. non-metastatic cell lines and metastatic vs. healthy mammary epithelium cell lines. The transcription factors EN2, NFE2L3 and SALL4, are upregulated in metastasis in both assessments. However, AR is upregulated in metastasis compared with the healthy mammary epithelium cell line, and genes such as EBF1 and LHX2 are upregulated in metastasis only when compared with the non-metastatic breast cancer cell line MDA-MB-468GFP.

We also assessed the differential expression of the metastasis influence genes in metastatic tumours compared with healthy tissue and non-metastatic tumours in MMTV-Wnt1 transgenic mice. Together with the expression data from cell lines, this data provides further validation of the participation in metastasis for 20 metastasis influence genes (see Table 2).

**Table 2:** Role of the metastasis influence genes on the metastasis regulatory network, their expression in models of metastatic breast tumours and their association with distant metastasis-free survival

Gene	Regulon size	Central in the metastasis regulatory network	Bow-tie core of the metastasis regulatory network	Expression in BC subtypes	Differentially expressed in metastatic tumours	Germline-somatic interaction	Associated with DMFS	Regulon associated with DMFS	Stemness phenotype	Implicated in metastasis
AR	5	yes		BL	cell line 2		–	NKI; METABRIC		[42, 79]
BACH2	3			BL, HER2, LUM, NL	mouse		–			
CALN1	NA			BL, HER2, LUM, NL	–			NA		
CDCA8	NA			BL, HER2, LUM, NL	cell line 1		NKI; METABRIC; UNT	NA	yes	[80, 81]
CLEC14A	NA	yes		BL, HER2, LUM, NL	–		–	NA		[82]
COL10A1	NA			BL, HER2, LUM, NL	cell line 2		VDX	NA	yes	[83–86]
COMP	NA			BL, HER2, LUM, NL	–	yes	TRANSBIG	NA	yes	[87, 88]
EBF1	13		yes	BL, HER2, LUM, NL	cell line 1; mouse	yes	–	MAINZ	yes	
EN1	3	yes		BL	cell line 1	yes	–	MAINZ		[89, 90]
EN2	2			BL, HER2, LUM, NL	cell line 1; cell line 2	yes	–	NKI; METABRIC		[91–93]
EXO1	NA			BL, HER2, LUM, NL	–		MAINZ; UNT	NA	yes	[94, 95]
FLI1	4			BL, HER2, LUM, NL	–		MAINZ; UNT	MAINZ; METABRIC; UNT; VDX		[96–98]
GNA14	NA			BL, HER2, LUM, NL	cell line 2	yes	–	NA		[99]
GPIHBP1	NA	yes		BL, HER2, LUM, NL	mouse		–	NA	yes	
GRM7	NA			BL, HER2, LUM, NL	–	yes	–	NA		
L3MBTL4	6		yes	BL, HER2, LUM, NL	–		UNT	–	yes	
LHX2	3			BL, HER2, LUM, NL	cell line 1		TRANSBIG; METABRIC	METABRIC		[100]
LRP1B	NA			BL, HER2, LUM, NL	–	yes	–	NA		[101–104]
LRRC4B	NA			BL, HER2, LUM, NL	–	yes	–	NA		
MEF2A	4			BL, HER2, LUM, NL	–	yes	–	TRANSBIG		[105]
METTL11B	NA	yes		BL, HER2, LUM, NL	–		–	NA	yes	
NEIL3	NA	yes		BL, HER2, LUM, NL	cell line 1		TRANSBIG; METABRIC	NA	yes	[106]
NEK2	NA	yes		BL, HER2, LUM, NL	cell line 1; mouse		NKI	NA	yes	[107, 108]
NFE2L3	3	yes		BL, HER2, LUM, NL	cell line 1; cell line 2		–	–		[109, 110]
NMNAT3	NA			BL, HER2, LUM, NL	–		–	NA		
NR3C1	5		yes	BL, HER2, LUM, NL	cell line 2; mouse	yes	–	NKI; TRANSBIG; METABRIC	yes	[111–113]
RP9P	NA			BL, HER2, LUM, NL	–		–	NA		
RPS6KA2	NA			BL, HER2, LUM, NL	–		–	NA		[114]
SALL4	6			BL, HER2, LUM, NL	cell line 1; cell line 2	yes	–	MAINZ	yes	[115,116]
SMAD3	3			BL, HER2, LUM, NL	cell line 1		–	–		[117–119]
SMARCD3	NA			BL, HER2, LUMB	–		–	NA		[120,121]
SMYD3	6		yes	BL, HER2, LUM, NL	cell line 1		–	–		[122–124]
SPARCL1	NA	yes		BL, HER2, LUM, NL	cell line 2		NKI	NA	yes	[125–127]
STARD8	NA	yes		BL, HER2, LUM, NL	–		–	NA		[128]
TMEM132C	NA			BL, HER2, LUM, NL	–		–	NA	yes	
TNS1	NA	yes		BL, HER2, LUM, NL	cell line 1; mouse		–	NA	yes	[129,130]
TSHZ2	28		yes	BL, HER2, LUM, NL	–	yes	–	MAINZ; NKI; METABRIC	yes	[35,131]
TUBA1C	NA			BL, HER2, LUM, NL	mouse		METABRIC	NA	yes	[132,133]
ZNF385D	3			BL, HER2, LUM, NL	–	yes	–	METABRIC	yes	

The gene is central in the metastasis regulatory network if its community centrality (a measure of its importance by the number of network communities to which the gene belongs) is in the top 20%. The transcription factor is in the bow-tie core if its bow-tie score is in the top 10%. Abbreviations: BL, basal-line; HER2, HER2-enriched; LUM, luminal; LUMB, luminal-B; NL, normal-like. Cell line 1, the gene is upregulated in the metastatic breast cancer cell line MDA-MB-468GFP vs. the poorly metastatic breast cancer cell line MDA-MB-468LN; cell line 2, the gene is upregulated in the metastatic breast cancer cell line MCF7 vs. the mammary epithelium cell line MCF10. Germline-somatic interaction, the gene harbours germline variations associated with somatic events. Stemness phenotype, the gene has an expression profile across the TCGA breast tumour samples significantly correlated with the mRNA stemness index (see Methods). Associated with DMFS, the gene is significantly more associated with DMFS than random genes in the indicated expression datasets. Implicated in metastasis reference of the studies that show how the gene participates in the molecular mechanisms of metastasis.

### Germline variants in metastasis influence genes are associated with somatic events in cancer genes

Carter et al., [3] leveraged genotype, clinical, copy-number variation, and somatic mutation data from TCGA to search for germline variants that either: (i) predict the tissue of origin of the tumour (i.e., cancer type across the 22 types compiled in the TCGA); or (ii) are associated with somatic events (i.e., both somatic mutations and somatic copy-number changes) in cancer genes. They found 232 genes harbouring germline variants that predict breast cancer and 364 genes with germline variants associated with somatic events in cancer genes.

Since the focus of Carter’s work is significantly different from ours, we do not expect a high coincidence between the germline variants associated with the origin of breast cancer or associated with somatic alterations in cancer genes and the germline variants associated with the susceptibility to develop metastasis we are looking for in this study. Nevertheless, we find substantial overlap between the genes that affect somatic events from Carter et al., and our metastasis susceptibility genes (see Table 2).

This result further supports the idea that germline variants work in collaboration with somatic alterations to promote tumour development and progress, as well as provide additional validation of the implication of our metastasis influence genes.

### Metastasis influence genes correlate with distant metastasis-free survival

To validate the role of the metastasis influence genes in modulating metastasis, we tested their involvement in breast cancer outcomes. Since metastasis determines the clinical outcome and survival of breast cancer patients, we used DMFS as a proxy to analyse the impact of our metastasis influence genes. We first investigated whether the expression of our metastasis influence genes was significantly altered in breast cancer patients and if their expression profiles were significantly more correlated with DMFS than random genes. We found that the association between the expression profile of a gene and the outcome is highly dependent on the cohort analysed and thus inconsistent among different gene expression datasets analysed. That means that a gene significantly associated with survival in a particular cohort will probably not be associated with survival in a different cohort. This instability and study-dependency of prognostic genes in cancer and its implication on the reliability of gene expression signatures have been studied before [40]. Venet et al., [41] showed that random gene signatures could be significantly associated with breast cancer outcome, being better outcome predictors than published signatures. We tested the 70-gene prognostic signature [5] in four gene expression data sets and found that it is significantly associated with breast cancer outcome in only two of them. This phenomenon has important implications for our work: since our metastasis influence genes do not result from analysing any gene expression dataset, they will compare unfavourably with random genes in any gene expression dataset. To provide an unbiased assessment of the association with DMFS our metastasis influence genes might have, we have tested them across several gene expression datasets (Supplementary Tables 3 and 4).

Table 2 reports the gene expression datasets in which our metastasis influence genes (or their regulons) are significantly associated with DMFS. Twenty out of 39 metastasis influence genes are associated with DMFS either by their expression or by the genes they regulate. Eleven of them are also upregulated in metastatic tumours. The androgen receptor (AR) activation regulates several pathways leading to different processes like proliferation, migration and invasiveness [42]. AR correlates with a good prognosis in ER-positive breast cancer patients and controls progression and drug resistance in ER-negative [43]. This agrees with our result that AR is upregulated in metastatic tumours, and its regulon is implicated in metastasis. The downregulation of Engrailed-2 (EN2) suppresses prostate cancer cell survival and metastasis [44], which is in concordance with our results, for EN2 is upregulated in metastatic breast cancer cell lines and regulates genes that influence metastasis. These are just two examples of how our metastasis influence genes can affect metastasis. Some of them were known to participate in metastasis, either in breast cancer or other tumours, as shown in Table 2. Approximately half of the metastasis influence genes participate in establishing the stemness phenotype in the tumour. Since cancer stem cells have a unique role in establishing the pre-metastatic niche [26, 45, 46], this result indicates that the metastasis influence genes could be involved in how the primary tumour prepares its future metastatic niche. Our analysis shows that the metastasis influence genes participate in metastasis not just because they are altered in the tumour but also because they have germline variants that make them prone to contribute to metastasis development.

In conclusion, our work moves onward with the seed and soil hypothesis—i.e., the host’s genetic background contributes to the development of metastasis. We unveiled several genes altered by germline variants that influence metastasis through their synergistic interaction with many other genes in our epistasis network. Therefore, we suggest that women who harbour specific sequence variants in the metastasis influence genes will deploy gene expression patterns that favour metastasis should they develop breast cancer.

The metastasis influence genes could affect the susceptibility to develop metastasis in two ways: either they are dysregulated in breast tumours and partake in the mechanisms of metastasis, or they regulate genes that form part of these mechanisms. Furthermore, they favour the dissemination of metastatic seeds and contribute to the congenial soil by priming the pre-metastatic niche.

## MATERIALS AND METHODS

### Patients and study design

We collected patients diagnosed in eight Spanish Hospitals that met the inclusion criteria. These criteria included female patients over 18 years old with histologically confirmed invasive breast cancer who had undergone surgery and had at least five years of follow-up. Patients with bilateral breast cancer and second primary tumours were excluded. All patients participating in the study gave their informed consent and protocols were approved by institutional ethical committees (Comité Coordinador de Ética de la Investigación Biomédica de Andalucía).

According to our extreme discordant phenotypes framework, we selected patients with a low risk of developing metastasis who nevertheless relapsed (good prognosis cases) and patients with a high risk of developing metastasis who did not develop a disseminated disease (poor prognosis cases). In the recently published 8^th^ Edition Cancer Staging Manual from the American Joint Committee on Cancer, patients with tumours smaller than 2 cm and without lymph node involvement have an excellent prognosis, independently of their biology. Likewise, tumours with more than ten positive lymph nodes (pN3) are classified as stage IIIC, independently of the primary tumour size, hormone receptor and HER2 status. These patients had a 5-year disease-free survival (DFS) of 30-39%, independently of the adjuvant treatment received and their oestrogen receptor (ER) status [39]; in this set of patients, the absence of relapse within five years was a rare event. Therefore, our cohort encompasses breast cancer patients that, regardless of their histological subtype, are either good prognosis cases, i.e., patients with tumours smaller than 2 cm and no lymph nodes affected who relapsed within five years after surgery; or poor prognosis cases, i.e., patients with more than ten lymph nodes affected regardless their tumour size who did not relapse within five years after surgery.

### Samples and genotyping

Genomic DNA was extracted from 3 mL of peripheral blood using the QiaAmp DNA Blood Mini Kit (Qiagen). The Human Genotyping Unit-CeGen CNIO conducted the genome-wide genotyping using the Illumina Infinium LCG Quad Assay protocol with the HumanOmni5-Quad Beadchip (Illumina). This chip contains ca. 4.3 million SNPs selectively distributed and separated by an average distance of 0.68 kb. The scanned signal raw intensities from all SNPs in the assay were analysed using the GenomeStudio software (Illumina). We filtered the data for quality control using the open-source tool PLINK [47]. We did not exclude any patient from the study due to low genotyping (call-rate < 90%). SNPs were excluded if they had a call-rate < 90% or a Hardy-Weinberg equilibrium *p*-value < 10^*−*6 ^.

### Epistasis network analysis

We looked for germline variants with a robust individual effect on susceptibility to metastasis (i.e., with a *p*-value < 5 × 10^*−*8 ^) by performing an association analysis between SNPs in the good and poor prognosis cases with the PLINK library within the Encore pipeline [48].

**Figure 3:**
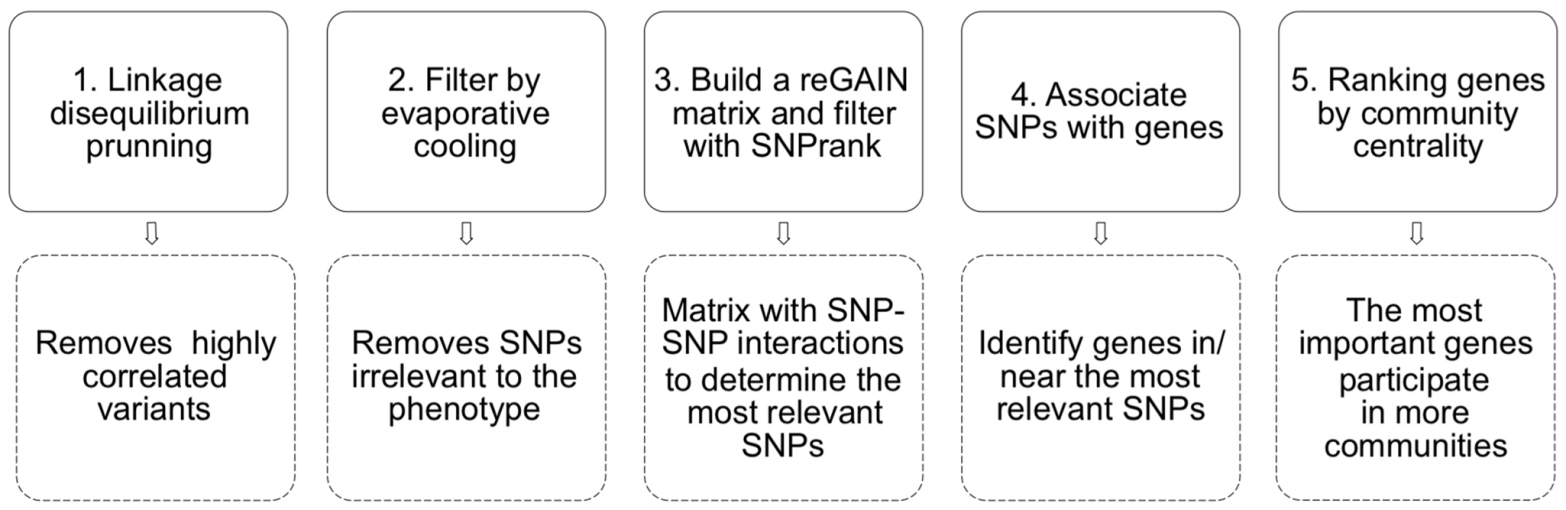
A pipeline of the epistasis network modelling with Encore. We used as input .bim/.bed/.bam files from PLINK. 1) The linkage disequilibrium pruning step removes highly correlated (i.e. low informative) SNPs. 2) Evaporative cooling is a machine learning method that integrates multiple importance scores while removing irrelevant genetic variants. In this step, we kept the 10000 most relevant SNPs, which constitutes a significant reduction from the initial ~ 4.3 million. 3) After filtering, Encore calculates the pairwise interaction for the 10000 SNPs with a generalised linear model. It computes a matrix of epistatic interactions among SNPs with Benjamini-Hochberg false discovery rate corrected *p*-values (reGAIN matrix). From that matrix, SNPs are ranked and filtered with SNPrank; we kept 2016 SNPs. 4) We obtained the names of the genes in or near (1 MB) the most relevant SNPs with the R library PostGWAS [50]. 5) Finally, we ranked the most relevant genes by their community centrality (using link communities [51]); genes are important if they participate in many communities.

We modelled and analysed a genetic interaction network from our genetic population data with the Encore pipeline. Figure 3 depicts the Encore workflow to model the epistasis network from genotyping data. Encore is an open-source tool for the analysis of biological data with the power to detect genetic variants relevant to a phenotype using genetic epistasis; it discovers variants without a substantial individual effect but whose relevance to the phenotype comes from their multiple interactions [49]. Encore focuses on common and rare variants to identify susceptibility hubs or groups of variants with numerous connections that influence the phenotype. To characterise these hubs, Encore computes a genetic association interaction matrix (reGAIN matrix) that ranks the variants according to their connection with other variants with the algorithm SNPrank [48]. Therefore, we obtain a list of ordered variants based on their importance to the phenotype of interest, which in our study is susceptibility to metastasis. With this list of SNPs and the reGAIN matrix, we identified the genes that harbour the most relevant SNPs (top SNPs). We modelled the gene epistasis network for the susceptibility to metastasis, keeping only significant epistatic interactions (Benjamini-Hochberg false discovery rate corrected *p*-value < 0.01).

According to network theory, a network revolves around a set of nodes termed central nodes. These central nodes capture the information flow represented in the network, and there are many ways to determine them [52]. In our gene epistasis network, central genes contain most of the metastasis susceptibility information through their interactions. To identify these central genes, we used the community centrality, which measures the importance of a gene by the number of network communities to which the gene belongs. We performed all the network analyses with the iGraph package [53] and the R platform for statistical computing.

### Gene expression in breast tumour samples, animal models and cell lines

We downloaded the RNASeq normalised gene expression dataset for breast cancer from the TCGA [54] with the R library RTCGAToolbox [55]. We transformed the RNASeq data to Z-scores so that per each tumour sample for each gene, we measured how many standard deviations (sd) away from the mean that gene expression is. We considered those with *Z* >1.96 (roughly *p*-value < 0.05 or more than 2 sd away) to be differentially expressed. We considered the tumour *t* and control *c* samples in the calculation of Z-scores for each gene *g* using the following equation:


$$Z_{t}^{g}=\frac{E_{t}^{g}-\langle E_{c}^{g}\rangle }{\sigma_{c}^{g}}$$

We thus compare the expression of gene *g* in the tumour sample *t* with the average and standard deviation of *g* in control samples. The gene’s tumour expression index is the proportion of tumour samples in which the gene is differentially expressed (i.e.) |*Z*| >1.96. We established a threshold for the tumour expression index using a random model of 10000 genes: less than 5% of random genes have a tumour expression index higher than 0.35. Therefore, we considered a gene expressed in tumour samples if its tumour expression index was higher than 0.35.

We performed differential expression analyses in animal models and breast cancer cell lines. We used microarray expression data from the Gene Expression Omnibus (GEO) dataset GSE84917 for MMTV-Wnt1 transgenic mice to compare the expression profiles of metastatic versus non-metastatic mammary tumours and metastatic mammary tumours versus healthy mammary tissue. We considered genes dysregulated if their logFC > 1 and their Benjamini and Hochberg false discovery rate (FDR) adjusted *p*-value < 0.01 with the limma 3.46 library on R 4.0.2 [56].

MDA-MB-468GFP is a poorly metastatic cell line; however, it has a variant (MDA-MB-468LN) with high metastatic ability. We compared the expression profiles of non-metastatic vs. metastatic tumours using the microarray expression data for these two cell lines in the GEO dataset GSE11683. We performed a differential expression analysis (logFC > 1, Benjamini and Hochberg FDR adjusted *p*-value < 0.01) with the limma library in R 4.0.2.

We also compared the expression profiles of metastatic tumours and healthy mammary epithelium using the cell lines MCF7 and MCF10A, respectively. MCF7 is a transformed breast cancer cell line derived from a metastatic site, and MCF10A is a normal-like mammary epithelial cell line. We performed a differential expression analysis (logFC > 1, Benjamini and Hochberg FDR adjusted *p*-value < 0.01) with the expression data from the GEO dataset GSE71862 [57], which contains RNA-seq data for these two cell lines. We calculated the differential gene expression using the DESeq2 version 1.30.1 [58] library in R 4.0.2.

### Map of gene regulation in metastasis

To represent the map of gene regulatory interactions in breast cancer metastasis, we have modelled a transcriptional regulatory network focused on metastasis. We started by building a broad gene regulatory network for breast cancer from the collection of 1612 transcription factors (TF) compiled in [59] and the genes controlled by those TFs, which we obtained from the TCGA (RNASeq breast cancer gene expression dataset). We used the RTN pipeline [60] to reconstruct transcriptional regulatory networks.

A transcriptional regulatory network consists of a collection of TFs and their regulated target genes. Each TF guides the expression of a set of genes, which forms a regulon. Therefore, TFs are regulators that either activate or repress the expression of the target genes. The RTN pipeline first computes the interactions between each TF and all potential target genes through the mutual information between a regulator and all potential targets—i.e., the mutual dependence between the expression profiles of the TF and their targets. Then, it performs a bootstrapping analysis to remove non-significant and unstable TF-target interactions. Each target gene may be linked to many TFs at this stage because regulation can occur through direct interactions between a transcription factor and a target gene and indirect interactions (TF-TF-target). The final step in the RTN pipeline is the ARACNe algorithm [61] to remove the weakest interaction in any triplet formed by two TFs and a common target gene, preserving the dominant TF-target pair.

From this broad network, we wanted to model a subnetwork centred on metastasis, that is, the part of the general network that contains the TFs and their regulons involved in the regulation of metastasis. Based on the idea that the network neighbourhood of a set of genes contains information about the biological processes in which the genes participate [62], we started from a set of genes involved in metastasis and characterised its network neighbourhood to obtain the gene regulatory network for metastasis in breast cancer.

We obtained the genes implicated in breast cancer metastasis from three sources: genes differentially expressed in metastasis samples, genes involved in the stemness phenotype, and genes dysregulated in metastasis through the metastasis expression index.

We compared the expression of metastatic samples (from both local and distant metastasis) with the expression of healthy control tissue from the TCG to obtain 65 differentially expressed genes (logFC ≥ 5; *p*-value < 0.0001). These genes were enriched in KEGG pathways related to metastasis, such as ECM-receptor interaction, IL-17 signalling and PPAR signalling.

Cancer stem cells are responsible for recurrence, relapse and metastasis [63]. Breast cancer metastasis involves acquiring stem-cell-like features characterised by the expression of markers that contribute toward a stemness phenotype [64]. Malta et al., [65] found that the stemness phenotype was generally most prominent in metastatic tumours and developed a stemness index for assessing this phenotype. We have used their mRNA stemness index and the weighted gene correlation analysis [66, 67] to find 75 genes whose expression profiles were significantly correlated with the stemness phenotype (*p*-value < 10^*−*10 ^). As expected, these genes are enriched in cell cycle-related biological processes and pathways related to metastasis and breast cancer progression, such as the oestrogen-responsive protein and Sonic Hedgehog signalling.

We developed the metastasis expression index analogous to the tumour expression index to obtain additional genes implicated in breast cancer metastasis. In this case, we obtained a Z-score for each gene in each breast metastasis sample from the TCGA RNASeq gene expression dataset by comparing the gene expression in the metastasis sample with the average and standard deviation of the gene expression in tumour samples. Thus, we obtained 121 genes dysregulated in metastatic breast tumours that were enriched in processes related to metastasis, such as the activation of epithelial cell proliferation and Wnt signalling.

We mapped the 261 genes related to metastasis onto the general breast cancer gene regulatory network. We used the DIAMOnD network diffusion algorithm [68] to obtain the network neighbourhood of these genes. DIAMOnD evaluates the significance of the connections that the initial set of genes has in the network to incorporate those genes better connected with the initial set. Therefore, with enough iterations of the algorithm (200 iterations), we obtained the subnetwork that comprises the initial set of 261 genes and their network neighbourhood, resulting in a gene regulatory network of breast cancer metastasis.

The metastasis regulatory network is modular. Each community of genes that interact more closely among them than with the rest of the network tends to encode a particular feature of the phenotype encoded in the network. We have highlighted those communities associated with metastatic processes through the over-representation test (multiple false discovery rate controlled; *q*-values < 0.001) on the Gene Ontology, REACTOME and the MSigDB hallmark gene set collection [69].

Gene regulatory networks often exhibit a bow-tie topology [70]. The presence of a robust interconnected core characterises the topology of these networks; this core is essentially a set of genes that communicate the fan-in component of source nodes (the transcription factors) with the fan-out component of sink nodes (i.e., the target genes). The core of the bow-tie structure reduces the number of genes and connections required to connect the transcription factors with the target genes, decreasing perturbation and noise [71]. We can identify the genes that belong to the core of the network by the bow-tie score [72]:


$$b\text{​ }(v)=\frac{S_{v}T_{v}}{S\;T}$$

Where *S_v_* is the number of source nodes (i.e., transcription factors) that can reach the gene *v*, *T_v_* is the number of target genes that *v* can reach, *S* and *T* are the total number of source and target nodes, respectively. We have implemented the bow-tie score to characterise the topology of the metastasis gene regulatory network.

### Survival analysis

We identified the transcription factors among our genes of interest and their regulons on our gene regulatory map. We tested the association of our genes and their regulons (when they are transcription factors) with breast cancer outcomes using the Kaplan-Meier survival analysis with the SigCheck R library [73]. For each gene and each regulon, we tested whether they were more significantly associated with distant metastasis-free survival (DMFS) than random genes (comparative survival analyses with random genes), on six gene expression datasets of breast cancer: NKI, 319 samples [5]; METABRIC, 1422 samples [74]; TRANSBIG, 198 samples [75]; MAINZ, 200 samples [76]; UNT, 125 samples [77]; and VDX, 344 samples [78]. The algorithm computes the mean expression value for each sample across the regulon (or the gene) in each independent survival analysis, which allows dividing the samples into a high expression group and a low expression group. Comparing the survival curves of these two groups results in a *p*-value that indicates the confidence that the samples are separable into groups with distinct survival outcomes. The comparative survival analysis produces an empirical *p*-value of the performance of genes or regulons against random genes in 1000 independent survival analyses.

## SUPPLEMENTARY MATERIALS




