# Supplementary material for: Role of germline variants in the metastasis of breast carcinomas
Source: Oncotarget. 2022 Jun 30;13:843–62. doi: 10.18632/oncotarget.28250 (PMC9245581; doi:10.18632/oncotarget.28250)
Supplement: Supplementary file 1 [file oncotarget-13-28250-s001.pdf]

Role of germline variants in the metastasis of breast carcinomas

SUPPLEMENTARY MATERIALS

Supplementary Table 1: Characteristics of patients and prognosis groups

Characteristic	Good prognosis (*N* = 34)	Poor prognosis (*N* = 63)	*p*-val
Age at diagnosis (years)			
Median	56	49	0.3227
Range	34–89	29–84	
Menopausal status			
Premenopausal	10 (29%)	34 (54%)	0.0354
Postmenopausal	24 (71%)	29 (46%)	
Histological type			
Ductal	28 (82%)	54 (86%)	0.0179
Lobular	2 (6%)	9 (14%)	
Others	4 (12%)	0	
Histological grade			
1	1 (3%)	4 (6%)	0.3370
2	20 (59%)	28 (45%)	
3	10 (29%)	27 (43%)	
NA	3 (9%)	4 (6%)	
Estrogen receptor			
Positive	23 (68%)	45 (71%)	0.7585
Negative	10 (29%)	15 (24%)	
NA	1 (3%)	3 (5%)	
Progesterone receptor			
Positive	17 (50%)	40 (63%)	0.2252
Negative	16 (47%)	20 (32%)	
NA	1 (3%)	3 (5%)	
HER2			
Positive	8 (23%)	9 (14%)	0.3947
Negative	22 (65%)	46 (73%)	
NA	4 (12%)	8 (13%)	
Histological subtype			
Luminal (HR+, HER2−)	17 (50%)	36 (57%)	0.5231
HER2+	8 (23%)	9 (14%)	
Triple negative (HR-, HER2−)	5 (15%)	10 (16%)	
NA	4 (12%)	8 (13%)	
Adjuvant chemotherapy			
Yes	14 (41%)	55 (87%)	<0.001
No	20 (59%)	7 (11%)	
NA	0	1 (2%)	

Adjuvant hormonotherapy			
Yes	18 (53%)	48 (76%)	0.0345
No	16 (47%)	15 (24%)	
Adjuvant radiotherapy			
Yes	22 (65%)	54 (86%)	0.0551
No	11 (32%)	9 (14%)	
NA	1 (3%)	0	

Abbreviations: NA: not available; HR: hormone receptor.

Supplementary Table 2: SNPs prioritised by SNPrank. See Supplementary Table 2

Supplementary Table 3: Kaplan-Meier survival analysis for the metastasis influence genes across six breast cancer gene expression datasets

Gene	Regulon size	KM random *p*-value					
		NKI	METABRIC	TRANSBIG	MAINZ	UNT	VDX
AR	5	0.115	0.549	0.784	0.951	0.536	0.81
BACH2	3	0.257	0.053	0.553	0.517	0.438	0.112
CALN1	NA	NA	0.439	NA	NA	0.374	NA
CDCA8	NA	0.005	0.001	0.098	0.242	0.079	0.133
CLEC14A	NA	NA	0.164	NA	NA	0.218	NA
COL10A1	NA	0.437	0.33	0.395	0.541	0.846	0.024
COMP	NA	0.534	0.464	0.011	0.114	0.979	0.873
EBF1	13	0.346	0.129	NA	NA	0.152	NA
EN1	3	0.291	0.656	0.385	0.776	0.418	0.139
EN2	2	0.234	0.488	0.238	0.801	0.76	0.775
EXO1	NA	0.001	0	0.032	0.005	0.256	0.304
FLI1	4	0.207	0.946	0.983	0.003	0.022	0.101
GNA14	NA	0.823	0.814	0.855	0.446	0.143	0.69
GPIHBP1	NA	NA	0.252	NA	NA	0.81	NA
GRM7	NA	0.902	0.702	0.111	0.164	0.71	0.862
L3MBTL4	6	NA	0.833	NA	NA	0.026	NA
LHX2	3	0.245	0.03	0.014	0.198	0.609	0.738
LRP1B	NA	0.617	0.364	0.292	0.121	0.931	0.585
LRRC4B	NA	NA	NA	NA	NA	0.538	NA
MEF2A	4	0.823	0.318	0.496	1	0.102	0.42
METTL11B	NA	NA	NA	NA	NA	NA	NA
NEIL3	NA	0.237	0.022	0.022	0.231	0.184	0.12
NEK2	NA	0.003	0.124	0.247	0.161	0.114	0.364
NFE2L3	3	0.156	0.872	0.464	0.904	0.971	0.862
NMNAT3	NA	0.147	0.13	NA	NA	0.754	NA
NR3C1	5	0.103	0.099	0.983	0.176	0.058	0.682
RP9P	NA	NA	NA	NA	NA	0.671	NA
RPS6KA2	NA	0.68	0.288	0.331	0.099	0.149	0.947
SALL4	6	0.467	0.051	NA	NA	0.735	NA
SMAD3	3	0.939	0.651	0.751	0.504	0.305	0.14
SMARCD3	NA	0.181	0.127	0.862	0.451	0.535	0.889

SMYD3	6	0.841	0.682	0.743	0.08	0.636	0.581
SPARCL1	NA	0.028	0.067	0.871	0.171	0.174	0.365
STARD8	NA	0.961	0.456	0.496	0.422	0.429	0.465
TMEM132C	NA	NA	0.522	NA	NA	0.736	NA
TNS1	NA	0.585	0.306	0.166	0.77	0.684	0.663
TSHZ2	28	0.329	0.184	0.539	0.424	0.416	0.725
TUBA1C	NA	NA	0.028	0.443	0.408	0.686	0.709
ZNF385D	3	NA	0.946	0.705	0.285	0.823	0.937

Supplementary Table 4: Kaplan-Meier survival analysis for the regulons of the metastasis influence genes across six breast cancer gene expression datasets

Gene	Regulon size	Regulon KM random *p*-value					
		NKI	METABRIC	TRANSBIG	MAINZ	UNT	VDX
AR	5	0.02	0.002	0.652	0.491	0.637	0.961
BACH2	3	0.276	0.377	0.379	0.218	0.269	0.955
EBF1	13	0.296	0.086	0.42	0.037	0.115	0.737
EN1	3	0.089	0.507	0.639	0.026	0.561	0.828
EN2	2	0.026	0.006	0.822	0.066	0.447	0.753
FLI1	4	0.141	0.035	0.616	0.004	0.006	0.035
L3MBTL4	6	0.899	0.916	0.411	0.188	0.55	0.345
LHX2	3	0.779	0	0.702	0.338	0.215	0.83
MEF2A	4	0.885	0.887	0.009	0.259	0.269	0.614
NFE2L3	3	0.321	0.917	0.459	0.22	0.781	0.127
NR3C1	5	0.004	0	0.023	0.293	0.107	0.639
SALL4	6	0.877	0.951	0.366	0.009	0.374	0.374
SMAD3	3	0.444	0.928	0.687	0.975	0.228	0.943
SMYD3	6	0.122	0.742	0.166	0.096	0.388	0.967
TSHZ2	28	0.045	0.07	0.55	0.03	0.512	0.435
ZNF385D	3	0.3	0.025	0.718	0.445	0.753	0.464
